# Modification of Graphene on Ultramicroelectrode Array and Its Application in Detection of Dissolved Oxygen

**DOI:** 10.3390/s150100382

**Published:** 2014-12-26

**Authors:** Jinfen Wang, Chao Bian, Jianhua Tong, Jizhou Sun, Yang Li, Wen Hong, Shanhong Xia

**Affiliations:** 1 State Key Laboratory of Transducer Technology, Institute of Electronics, Chinese Academy of Sciences, Beijing 100190, China; E-Mails: cbian@mail.ie.ac.cn (C.B.); jhtong@mail.ie.ac.cn (J.T.); sunjizhou628@163.com (J.S.); math101@163.com (Y.L.); shxia@mail.ie.ac.cn (S.X.); 2 National Key Laboratory of Microwave Imaging Technology, Institute of Electronics, Chinese Academy of Sciences, Beijing 100190, China; E-Mail: grad.mitl@mail.ie.ac.cn

**Keywords:** graphene, ultramicroelectrode array, dissolved oxygen, microsensor, MEMS

## Abstract

This paper investigated two different modification methods of graphene (GN) on ultramicroelectrode array (UMEA) and applied the GN modified UMEA for the determination of dissolved oxygen (DO). The UMEAs were fabricated by Micro Electro-Mechanical System (MEMS) technique and the radius of each ultramicroelectrode is 10 μm. GN-NH_2_ and GN-COOH were modified on UMEA by using self-assembling method. Compared with GN-NH_2_ modified UMEA, the GN-COOH modified UMEA showed better electrochemical reduction to DO, owing to better dispersing and more active sites. The GN-COOH on UMEA was electroreduced to reduced GN-COOH (rGN-COOH) to increase the conductivity and the catalysis performance. Finally, the palladium nanoparticles/rGN-COOH composite was incorporated into DO microsensor for the detection of DO.

## 1. Introduction

Dissolved oxygen (DO) is a vital parameter in water monitoring. A number of biological reaction and chemical reaction in water are influenced directly or indirectly by the quantity of DO. The concentration of DO in water below 2 mg/L for some hours can cause large fish kills [1]. The determination of levels of DO is critical to the water quality. The measurement of DO with suitable sensors shows many advantages over typically chemical titrations, such as simple, portable and time-saving. Therefore, development of DO sensor devices is very important for measuring molecular gaseous oxygen content in environmental water [2]. Various optical sensors [3–6] and electrochemical sensors [7–10] are investigated for the detection of DO. The optical DO sensors include fluorescent sensors [3,4], electrochemiluminescence sensors [5] and luminescence sensors [6]. Chu *et al.* [3] investigated an optical fiber DO sensor based on Pt(II) complex and core-shell silica nanoparticles incorporated with sol-gel matrix for fluorescence detection of DO. The optical fiber DO sensor showed high sensitivity because of increased surface area and penetration of substantial amount oxygen on porous silica. Zheng *et al.* [5] investigated a hot electron induced cathodic electrochemiluminescence at a disposable CdS modified screen printed carbon electrodes for the detection of DO. This sensor showed low detection limit, good stability and was satisfactorily reproducible, showing potential application in DO and biochemical oxygen demand detection. Three types of electrochemical sensors have been reported for DO detection, amperometric sensors [7,8], galvanic oxygen sensors [9] and potentiometric sensors [10]. Martin *et al.* [8] developed an amperometric sensor for determination of DO by nickel-salen polymeric film modified electrode. The proposed sensor can be used for the quality control and routine analysis of DO in commercial samples and environmental water. Zhuiykova *et al.* [10] investigated an antifouling of potentiometric solid-state DO sensors based on nanostructured Cu_0.4_Ru_3.4_O_7_ + RuO_2_ sensing electrodes. The sensor can be utilized as an effective measuring tool for analysis and control of the DO level in the complex fresh water environments. Among the mentioned principles, the most commonly used DO sensors are typically a Clark-type cell, which contains an electrode system including a selective membrane. The response current of the DO sensor is the oxygen reduced current at the cathode after the oxygen diffusing through the membrane. DO sensors presently reported are relatively large devices [5,8,10] which are difficult to use in wireless sensor network for environmental monitoring. Miniaturization has been an important trend in fabrication of sensors. Micro Electro-Mechanical System (MEMS) provides the possibility of easy and cost effective production of smaller devices.

The outstanding characteristics of ultramicroelectrode array (UMEA) are resulted from the radial diffusion. When the center-to-center separation is large enough to avoid the “shielding” effect, the radial diffusion is dominant and the maximum current density is achieved [11]. UMEA has the merits of fast mass-transport, low ohmic drop, high signal-to-noise ratios and satisfied current signal [12]. Therefore, UMEA is an important tool in electrochemical sensor fabrication, which possesses high sensitivity, low limit of detection, fast response time and well reproducible geometries [13]. However, with minimizing the dimension of UMEA, the modification methods are limited.

Graphene (GN) has several advantages, such as huge surface area, high conductivity and fast electron transfer [14]. These unique characteristics enable it to hold great promise for application in many fields, especially for developing high-performance sensors [15,16]. GN has been demonstrated to show excellent electrochemical catalytic activities toward several species, such as oxygen [17], small molecules [18], heavy metals [19] and so on. In sensor modification, the common method of GN modification is drop-casting solution [20,21]. This method is not suitable for UMEA modification. Because the drop-casting solution-based GN can make the insulation of UMEA electric, the unique characteristics of UMEA is lost. Self-assembled method is based on thiols and related molecules, which can modify the founctionalized GN only on gold interface [22]. In addition, founctionalized GN are easier to disperse in water or organic solvents, which can improve the dispersion and homogeneity of GN. Due to the functional groups presented in chemical derived GN and the inevitable carbon-vacancy defect [23], metal ions can be induced into GN sheets to form stable composite materials. In addition, physical properties such as high surface area and mechanical strength make GN an ideal material to be utilized as a catalyst support [24]. Palladium gains much attention in electrocatalysts for oxygen reaction, because of similar properties to platinum, more abundant on the earth and less expensive than platinum [25]. Incorporation the advantages of Pd and GN, sensors based on Pd/GN composites show potential application in electrocatalysis of oxygen and other species. Seo *et al.* [26] investigated the electroreduction of oxygen on GN-supported Pd nanoparticles and an excellent oxygen reduction reaction activity was observed. Rajkumar *et al.* [27] modified highly loaded palladium nanoparticles decorated chemically reduced graphene oxide on glassy carbon electrode and investigated its electrocatalytic applications in dopamine and diclofenac.

In this paper, GN-NH_2_ and GN-COOH were self-assembled on gold ultramicelectrode by covalently bonding of two thiols compounds (mercaptopropionic acid and mercaptoethylamine). The characteristics of GN-NH_2_ and GN-COOH modified UMEAs were investigated by electroreducing of DO. The GN-COOH modified UMEAs were further electroreduced and modified by palladium nanoparticles (PdNPs) for DO microsensor fabrication.

## 2. Experimental Section

### 2.1. Reagents and Apparatus

GN-NH_2_ and GN-COOH (carboxyl ratio >5.0 wt.%) were obtained from Nanjing XFNANO Materials Tech Co., China. The *N*-(3-Dimethylaminopropyl)-*N*′-ethylcarbodiimide (EDC), *N*-Hydroxy-succinimide (NHS), palladium nitrate, mercaptopropionic acid and mercaptoethylamine were purchased from Sigma and Aldrich. Teflon membrane was purchased from Sartorius, Germany. All other chemicals were of analytical grade. Deionized water was used throughout the experiment. Different DO solutions were prepared by Na_2_SO_3_ solutions. DO concentration was detected by commercial DO meter (Eutech CyberScan DO110 Dissolved Oxygen Meter, Vernon Hills, IL, USA). Scanning electron microscope (SEM) analysis was carried out on S-4800 field emission scanning electron microscope produced by Hitachi (Tokyo, Japan). Raman spectra were obtained on an AvaRaman-532TEC (AVANTES B.V., Eerbeek, Holland) employing a 532 nm laser beam. Electrochemical experiments were performed on Gamry Reference 600 electrochemical measurement system (Gamry Instruments Co., Ltd., Warminster, PA, USA) with a three-electrode cell. The cell consisted of a modified UMEA working electrode, a commercial saturated Ag/AgCl (CRE) or on-chip Ag/AgCl electrode (ORE), and an on-chip Pt counter electrode. The reference electrode in the DO microsensor was ORE, and the others were CRE.

### 2.2. UMEA Fabrication

**Figure 1.**
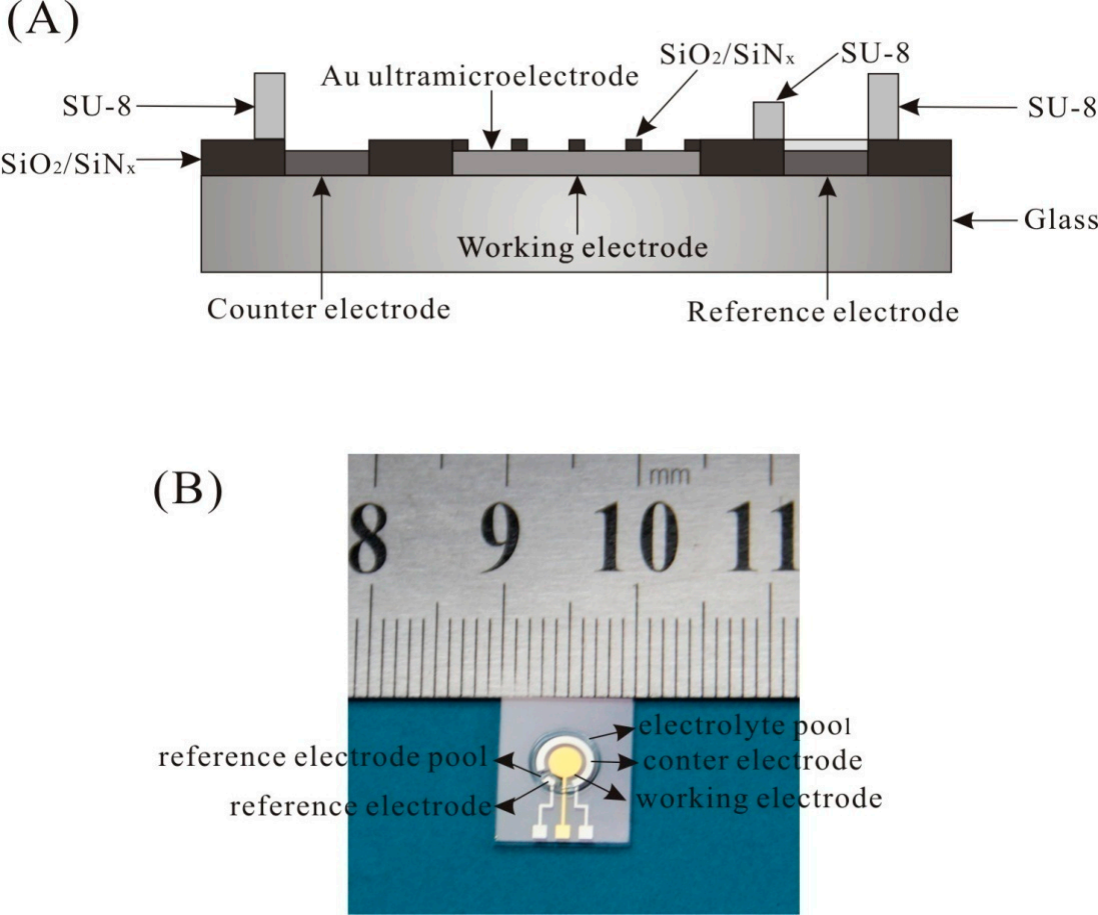
The cross-sectional view (**A**) and the photo (**B**) of ultramicroelectrode array (UMEA) chip.

The UMEA chip was fabricated by MEMS technique on glass. The cross-sectional view of the UMEA chip is shown in Figure 1A. It contains an array of Au working ultramicroelectrode, a Pt counter electrode, and a Pt electrode. The working electrode consists of 112 disk-shaped Au ultramicroelectrode and the insulating layer is SiO_2_/SiN_x_. The radius of disk-shaped ultramicroelectrode is 10 μm, and the interelectrode spacing is 200 μm. The active area of UMEA is 0.035 mm^2^. SU-8 with different height was fabricated to form the reference electrode pool and the electrolyte pool. The photo of the UMEA chip is shown in Figure 1B and the size of the UMEA chip is about 1 × 1.2 cm. The ORE was made by coating Ag/AgCl ink on the Pt electrode, and then annealing at 90 °C for an hour.

### 2.3. UMEA Modification

Exactly 10 mM of mercaptopropionic acid (HSCH_2_CH_2_COOH) solution was added on the surface of UMEA for 12 h at 4 °C. The surface of the modified electrode was washed with ethanol and deionized water successively, and then dried in the air. Because of the strong Au-S bond, HSCH_2_CH_2_COOH molecular were adsorbed on the active sites of UMEA. Then a mixed solution of EDC and NHS was applied onto the surface of HSCH_2_CH_2_COOH modified UMEA for 15 min at 4 °C to active the carboxylic group. Subsequently, the solution of GN-NH_2_ was applied onto the above electrode for 5 h at 4 °C and washed with deionized water. This step makes the carboxyl groups on the UMEA bonded with the amino group of GN-NH_2_ to form GN-NH_2_/UMEA.

**Figure 2.**
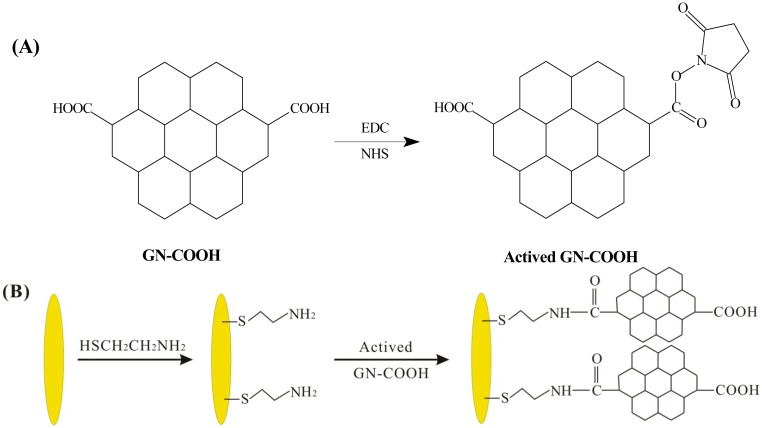
The scheme of active carboxylic group (**A**) and the modification process of GN-COOH on UMEA (**B**).

Exactly 10 mM of mercaptoethylamine (HSCH_2_CH_2_NH_2_) solution was added on the surface of UMEA for 12 h at 4 °C. A mixed solution of NHS and EDC was applied into the GN-COOH solution to active the carboxylic group. The process of the active carboxylic group is shown in Figure 2A. The above mixed solution was added on the surface of HSCH_2_CH_2_NH_2_ modified UMEA to form GN-COOH/UMEA. The scheme of the GN-COOH modified UMEA is shown in Figure 2B. The GN-COOH/UMEA was electrochemical reduced in PBS by cyclic voltammetry from 0 to −1.1 V at scan rate 50 mV/s to form rGN-COOH/UMEA. Finally, PdNPs were modified on rGN-COOH/UMEA by electrodeposition of 1 mM palladium nitrate solution at potential of −0.3 V for 5 s.

### 2.4. Microsensor Design

**Figure 3.**
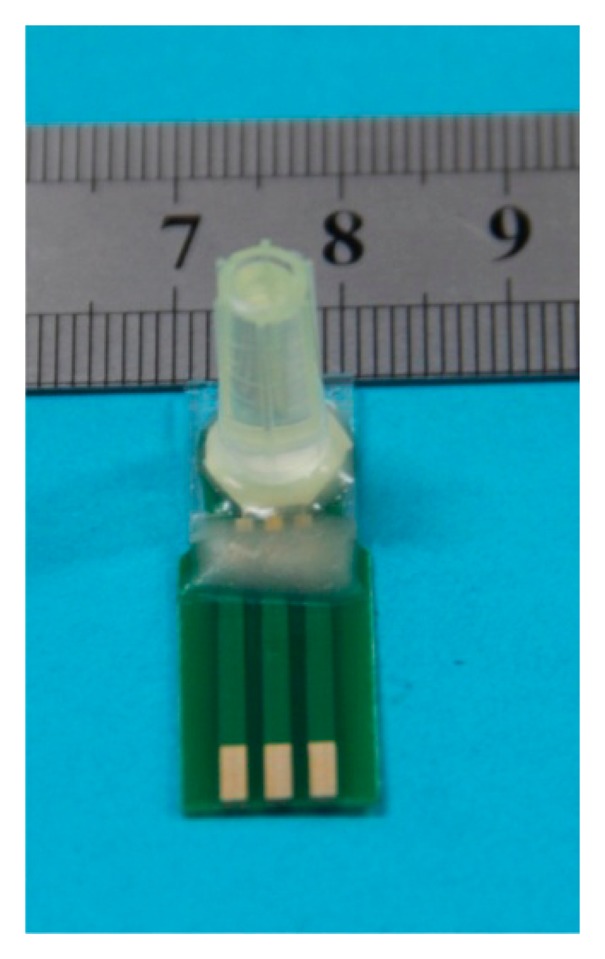
The photo of packaged dissolved oxygen (DO) microsensor.

The microsensor contains a three-electrode system, a reference electrode pool, an electrolyte pool, and an analyte pool. The working electrode is PdNPs/rGN-COOH/UMEA. The counter electrode is Pt counter electrode. The reference electrode is ORE. The electrolyte pool around the three-electrode system was covered with 0.1 M LiCl solution, and then a gas permeable membrane of Teflon was covered on the electrolyte pool. Finally, the analyte pool was made by fixing a plastic pipe on Teflon. The photo of packaged DO microsensor is shown in Figure 3.

## 3. Results and Discussion

### 3.1. Two Modification Methods of Graphene on UMEA

**Figure 4.**
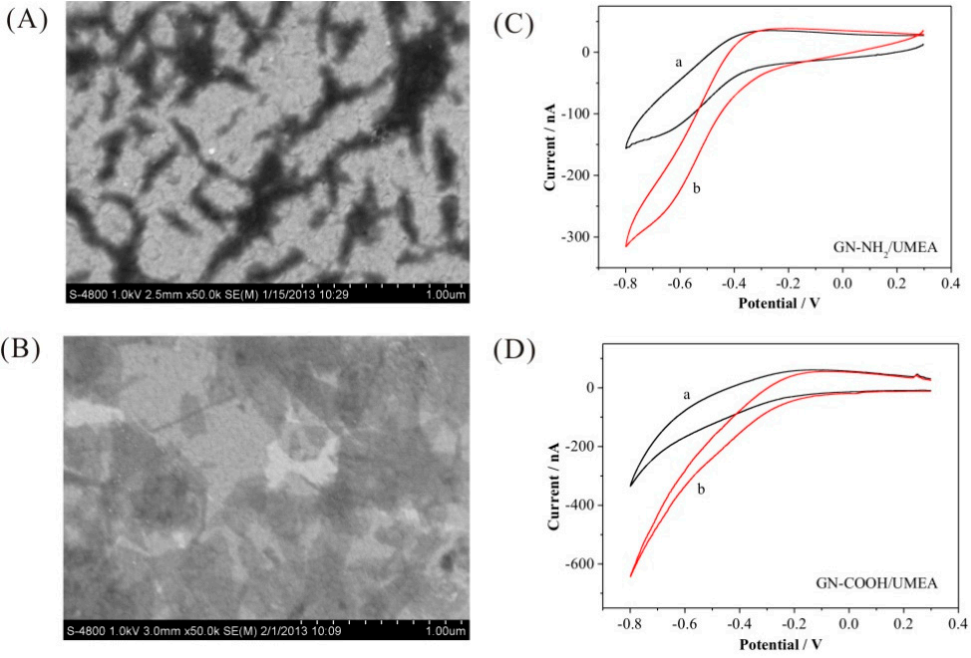
SEM images of GN-NH_2_ (**A**) and GN-COOH (**B**); Response of GN-NH_2_/UMEA (**C**) and GN-COOH/UMEA (**D**) in absence (a) and presence (b) of DO.

The surface morphology of GN-NH_2_ and GN-COOH on UMEA were compared in Figure 4A, B. The GN-NH_2_ aggregated and sporadically distributed on the gold surface. However, the GN-COOH is a sheet and more uniformly dispread on the gold surface, owing to the better solubility in water. The electroreduction ability of GN-NH_2_/UMEA and GN-COOH/UMEA to DO was shown in Figure 4C, D. The GN-NH_2_/UMEA showed reduction potential from −0.22 V, and the response current at −0.4 V is 31 nA. The GN-COOH/UMEA showed reduction potential from −0.15 V, and the response current at −0.4 V is 70 nA. Compared with GN-NH_2_/UMEA, the GN-COOH/UMEA has better electrocatalysis activity, showing lower reduction potential and higher response current. Because the GN-NH_2_ is enwrapped by polymers, the activity sites on the edge are decreased. In a further experiment, the GN-COOH/UMEA was adopted as the working electrode.

### 3.2. Electroredcution of GN-COOH to rGN-COOH on UMEA

**Figure 5.**
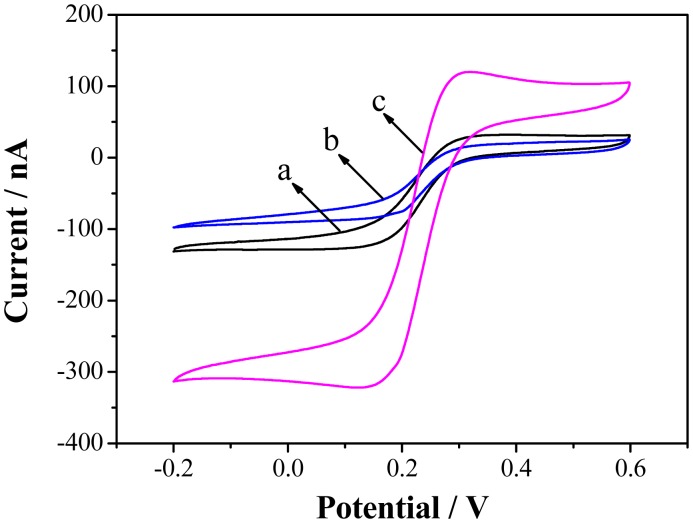
CV curves of bare UMEA (a), GN-COOH/UMEA (b) and rGN-COOH/UMEA (c) in 0.5 mM Fe(CN)_6_^3−^ solution.

GN-COOH is rich in oxygen-containing functional group, which affects the catalytic activity of GN [28]. In order to increase the conductivity of catalyst, reduction of GN-COOH to reduced GN-COOH is a key step. Electrochemical reduction of GN-COOH has drawn great attention due to its fast and green nature [29]. Electrochemical reduction of GN-COOH was carried out by cyclic voltammetry from 0 to −1.1 V at 50 mV/s. As shown in Figure 5, the CV curves of UMEA (curve a), GN-COOH/UMEA (curve b) and rGN-COOH/UMEA (curve c) were characterized in 0.5 mM Fe(CN)_6_^3−^ solution. A typical sigmoidal curve is obtained on the UMEA, showing a radial diffusion mode. Compared with the curve of UMEA, a decrease is observed in GN-COOH/UMEA, owing to the insulating property of the GN-COOH. After reduction of GN-COOH, a large increase is observed on the rGN-COOH/UMEA. The results showed that the GN-COOH has been reduced to rGN-COOH, which has good conductivity and fast electron transfer.

**Figure 6.**
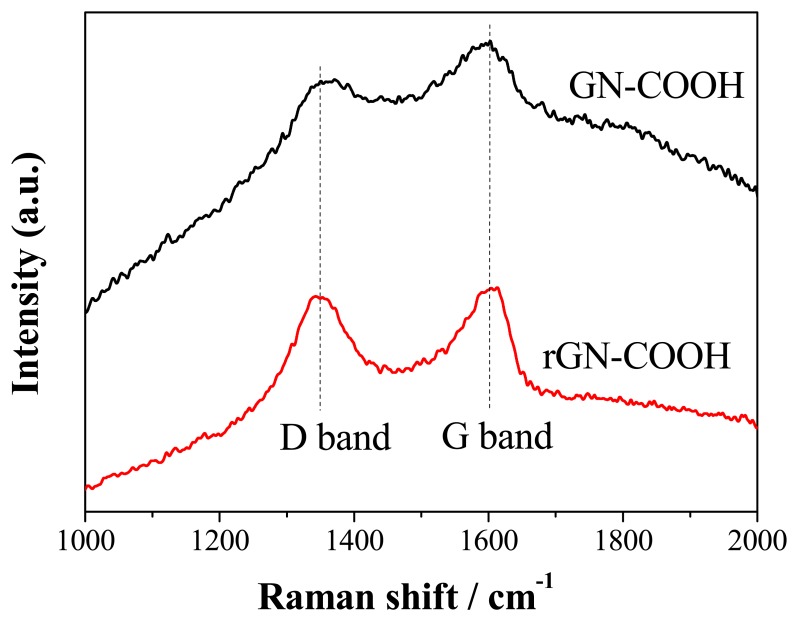
Raman spectra of GN-COOH and rGN-COOH.

Raman scattering is strongly sensitive to the electronic structure and proved to be an essential tool to characterize GN materials. The Raman spectrum of GN sheets is characterized by two main features. The D bands correspond to sp^3^-hydridized carbons and signify the presence of defects in GN, while the G bands correspond to sp^2^-hybridized carbons indicative of the ordered state in GN. The Raman spectra of GN-COOH and rGN-COOH are shown in Figure 6. The GN-COOH and rGN-COOH sheets display two prominent peaks at 1345 cm^−1^ and 1602 cm^−1^, corresponding to D bands and G bands, respectively. The D/G ratio intensity increased from GN-COOH to rGN-COOH, inferring that more defects form when some oxygen atoms are removed [30].

**Figure 7.**
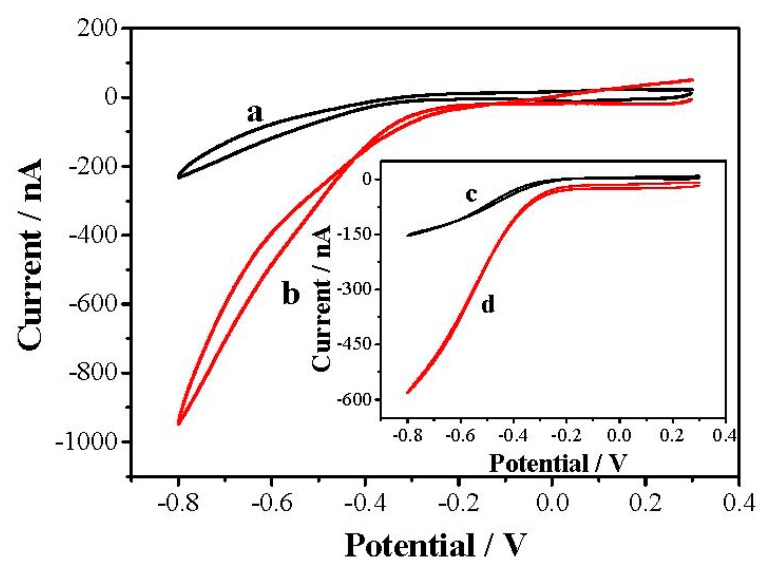
Response of rGN-COOH/UMEA in absence (a) and presence (b) of DO. The inset shows the response of bare UMEA in absence (c) and presence (d) of DO.

The catalytic activity of rGN-COOH/UMEA and bare UMEA are shown in Figure 7. The bare UMEA showed reduction potential from −0.2 V, and the response current at −0.4 V is 84 nA. The rGN-COOH/UMEA showed reduction potential from −0.15 V, and the response current at −0.4 V is 114 nA. Compared with the bare UMEA, the rGN-COOH/UMEA showed better catalytic activity toward DO. Upon comparison to the current response of GN-COOH/UMEA observed previously in Figure 4D, the response current of rGN-COOH/UMEA increased significantly. It is clear that a higher activity towards DO was obtained after electroreduction of GN-COOH. The reason for this may be that the carbonyl terminating groups in GN-COOH are high and hence potentially inhibit electron transfer [24]. After electroreduction, an improved electrochemical response resulted from fewer oxygenated species and more edge plane like-defects on rGN-COOH [24].

### 3.3. The Electrocatalysis of PdNPs/rGN-COOH to DO

**Figure 8.**
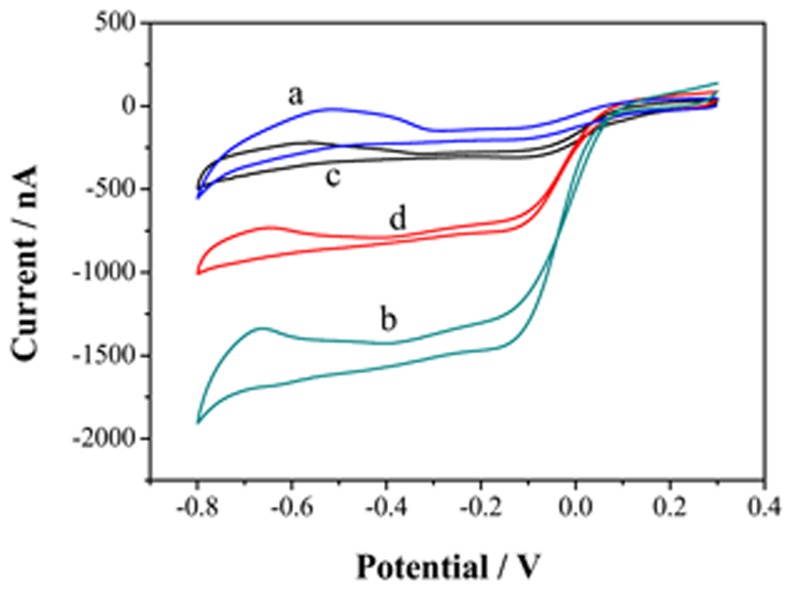
Response of PdNPs/rGN-COOH/UMEA in absence (a) and presence (b) of DO and PdNPs/UMEA in absence (c) and presence (d) of DO.

The further modification of reduced graphene oxide with metal NPs can increase its electrical conductivity to a large extent [31]. The formed defects in rGN-COOH may act as a nucleating center of the metal nanoparticles [23]. In addition, the rGN-COOH can act as a support to stabilize metal NPs [32]. Incorporation of catalyst particles onto rGN-COOH with good distribution can provide greater versatility in sensing process [32]. Thus, PdNPs was electrodeposited on rGN-COOH/UMEA to increase the electrocatalysis activity. The electrocatalytic properties of PdNPs/rGN-COOH/UMEA and PdNPs/UMEA in N_2_-saturated (Curves a and c) and air-saturated (Curves b and d) LiCl solution are shown in Figure 8. Compared with PdNPs/UMEA, the PdNPs/rGN-COOH/UMEA has higher electroreduction response, showing electroreduction potential from 0.1 V and response current of 1343 nA at the potential of −0.4 V. Additionally, in comparison to rGN-COOH/UMEA response (see Figure 7), the PdNPs/rGN-COOH/UMEA had more positive reduction potential and higher reduction current, showing better electrocatalysis performance to DO. The high activity of PdNPs/rGN-COOH/UMEA towards DO could be attributed to the combination effect of the high catalytic activity of PdNPs and rGN-COOH.

### 3.4. The Response of Microsensor to DO

**Figure 9.**
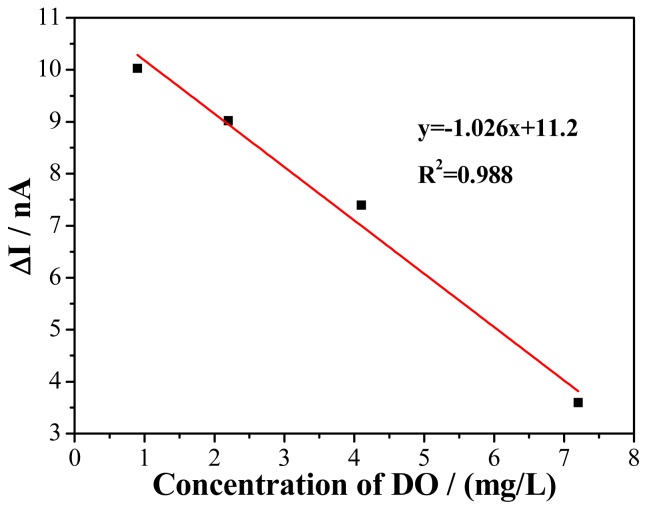
The linear range of DO microsensor.

The DO microsensor characteristics were verified by chronoamperometry method at the potential of −0.4 V (*vs.* ORE). The responses of chronoamperometry were recorded at different concentration of DO in solution. The linear range of DO microsensor is shown in Figure 9. The current difference (ΔI) between the current of 5 min (I_5_) and the current of 0 min (I_0_) is adopted as the current response. With the increasing concentration of DO from 0.9 to 7.2 mg/L, the response current was plotted against the DO concentration resulting in a straight line. The linear equation is ΔI (nA) = −1.026C (mg/L) + 11.2, with correlation coefficient of 0.988 and sensitivity of 1.026 nA/(mg/L). This behavior permits the use of this microsensor to monitor DO in solution.

## 4. Conclusions

Two different GN were modified on the UMEA by using self-assembling method. The surface morphology and the electroreduction ability of GN-NH_2_ and GN-COOH on UMEA were compared. The GN-COOH/UMEA showed better distribution and electroreduction ability. The GN-COOH was electroreduced to rGN-COOH to increase the electron transfer and the electroreduction activity. Then the PdNPs were electroreduce on rGN-COOH to further increase the electroreduction ability to DO. The PdNPs/rGN-COOH/UMEA as working electrode was constructed into DO micosensor. The microsensor showed a satisfying performance of DO and can be further used for detecting of DO in environmental water.
